# Synthesis of stable ACC using mesoporous silica gel as a support

**DOI:** 10.1186/1556-276X-9-450

**Published:** 2014-08-29

**Authors:** Fen Fu, Liang Guang Tian, Sheng Xu, Xian Gang Xu, Xiao Bo Hu

**Affiliations:** 1 State Key Laboratory of Crystal Materials, Shandong University, Shanda Nanlu Rd No.27, Jinan, Shandong Province 250100, China; 2 Shandong Institute of Supervision and Inspection on Product Quality, Jinan 250100, China

**Keywords:** Amorphous materials, Nanoparticles, Silica gel, Calcium carbonate

## Abstract

Stable amorphous calcium carbonate supported by mesoporous silica gel was successfully synthesized. The silica gel support is prepared through the hydrolytic polycondensation of ethyl silicate under suitable conditions. Laser scanning confocal microscopy (LSCM) observations reveal that the morphology of the products is branched with cruciform-like and flower-like structure. Raman spectroscopic analysis and scanning electron microscopy (SEM) observation of the products confirm the combination of stable amorphous calcium carbonate (ACC) nanoparticles and mesoporous silica gel. A possible growth mechanism for the branched structure has been proposed. Results indicate potential application of this work to ACC storage, crystal engineering, biomimetic synthesis, etc.

## Background

Amorphous calcium carbonate (ACC) has attracted increasing interest as a result of its potential use in biomimetic and industrial applications. However, it is a transient precursor phase to crystalline modification [1-4], so it is difficult to obtain in vitro. Stabilizing amorphous precusors is one of the major issues in biomineralization studies [5]. Moreover, people had been trying to add process-directing agents during the nucleation stage. Additives such as phosphorproteins [6], aspartic acid [7], and ployacrylic acid (PAA) [5] have been proved to act as stabilizers for ACC. In addition, researchers have also tried other inorganic substances, with the result that spherical ACC accompanied by vaterite or calcite was obtained [8].

The reason ACC is unstable under ambient conditions is because of its large interfacial energy. Accordingly, it would be interesting to develop a highly porous support material which would make it possible to lower the interfacial energy of ACC during the reaction [9]. With this in mind, silica gel was chosen as the material because of its tunable porosity via hydrolytic polycondensation of liquid precursors such as the silicon alkoxides under controlled conditions [10]. The first synthesis of porous silica was described by Kistler in 1931 [11]. Since that time, silica gels have been used as functional materials with an impressive range of applications [12]. The use of silica gel for CaCO_3_ single crystal growth has been employed as a means to control the purity and morphology [13,14]. However, a silica gel-based system for controlling the formation of amorphous CaCO_3_ has not been studied.

In this work, we used a porous silica gel support to form ACC for the first time. Silica gel is obtained through the hydrolytic polycondensation of ethyl silicate as an additive to a solution of CaCl_2_ and (NH_2_)_2_CO. The morphology of silica gel can be tailored to form a 3D-matrix during hydrolytic polycondensation under suitable conditions [9], so that support is afforded that lowers the interfacial energy of the ACC. The structure and morphology of the product were characterized by laser scanning confocal microscopy (LSCM), micro-Raman spectroscopy, and scanning electron microscopy (SEM).

## Methods

The ethyl silicate (ES), calcium chloride dihydrate (CaCl_2_··2H_2_O), urea, ethyl alcohol (C_2_H_5_OH), and sodium hydroxide (NaOH) used as precursors were of analytical grade and used without further purification. All chemicals were purchased from Sinopharm Chemical Reagent Co., Ltd (Shanghai, China). Deionized water with an electrical conductivity of less than 10^6^ S m^-1^ was taken from a Mili-Q system.

Four separate silica solutions were prepared by mixing 0.2 mL ethyl silicate, 0.2 mL alcohol, 6.5 mL NaOH (0.1 M), and deionized water in 100-mL plastic beakers and stirring for 1 h. A 0.5 M calcium chloride solution and 2.5 M urea solution were prepared in 30-mL quantities. Subsequently, different amounts (0.5, 1, 1.5, and 2 mL) of the 0.5 M calcium chloride solution and 1.5 mL of the 2.5 M urea solution were added to the plastic beakers. As a result, the concentration of CaCl_2_ is, respectively, 2.5, 5, 7.5, and 15 mM, in these four mixing solutions. Deionized water was added until the total amount of mixture was 100 mL. After that, 5 mL each of the solutions was transferred to separate Petri dishes, each with a 5 cm × 5 cm slide substrate. Each Petri dish was sealed by parafilm with seven pinholes and then incubated at 60°C until bakeout. The sample on the slide substrate was then subjected to analysis.

Laser scanning confocal microscopy (LSCM) and scanning electron microscopy (Hitachi S-4800 SEM, Hitachi, Ltd., Chiyoda-ku, Japan) were used to observe the morphology of the sample. SEM images were obtained without gold coating in order to avoid spurious results. The Raman scattering spectrum was obtained using the 532-nm line of a Nd, YAG laser as the excitation source. For all measurements with visible excitation, the slits were set at 100 μm and a × 100 objective was used.

## Results and discussion

**Figure 1:**
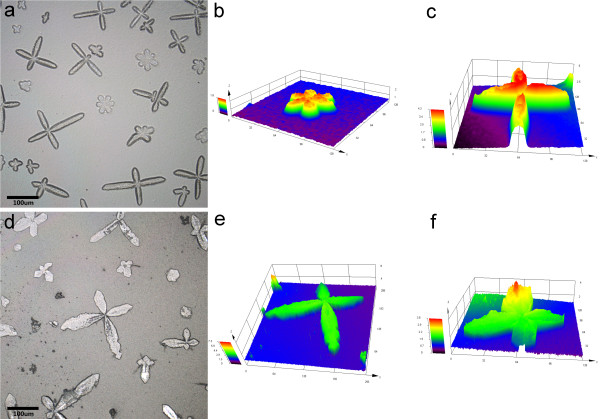
**LSCM images of branched products. (a)** obtained from 7.5 mM CaCl_2_; **(b)** high-magnification of cruciform-like product of **(a)**; **(c)** high-magnification of flower-like product of **(a)**; **(d)** obtained from 5 mM CaCl_2_; **(e)** high-magnification of cruciform-like product of **(d)**; **(f)** high-magnification of flower-like product of **(d)**.

Figure 1 shows the recorded LSCM images of the samples grown in the mixing solutions with CaCl_2_ concentrations of 7.5 mM (Figure 1a,b,c) and 5 mM (Figure 1d,e,f). The branched samples, including cruciform-like and flower-like structures are formed by varying the CaCl_2_ concentration from 5 to 7.5 mM. However, no such branched products are formed with a CaCl_2_ concentration that is less than 5 mM or greater than 7.5 mM (see Additional file 1: Figure S1). That is to say, the suitable CaCl_2_ concentration for the formation of branched products ranges from 5 to 7.5 mM. Note that the shape of the branched samples obtained with 7.5 mM CaCl_2_ (Figure 1a) is more pronounced than that obtained with 5 mM CaCl_2_ (Figure 1d). The magnified 3D contour maps shown in Figure 1b,c and Figure 1e,f further confirm the foregoing evidence that the aspect ratio of the branched product obtained with 7.5 mM CaCl_2_ (0.10 ~ 0.21) is lower than that obtained with 5 mM CaCl_2_ (0.05 ~ 0.15). One can conclude that the nature of the final products tends to be related to the CaCl_2_ concentration, where 7.5 mM appears optimal for forming the branched form.

**Figure 2:**
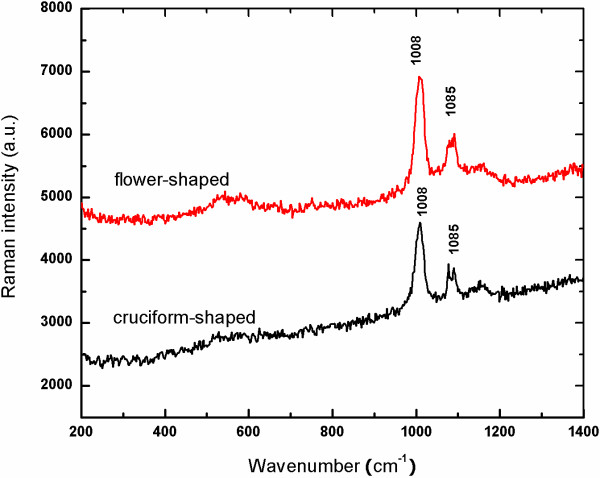
Micro-Raman spectra of branched products.

Figure 2 shows the Raman scattering spectra of the branched samples. Scattering bands centered at 1,008 and 1,085 cm^-1^ are seen for both the cruciform-like and flower-like samples. So, the branched samples, either cruciform-like or flower-like, are made of the same material. The peak at 1,085 cm^-1^ corresponds to the ν1 symmetric vibrational mode of the carbonate ion (CO_3_^2-^) in CaCO_3_[15-17]. The Raman spectrum of the branched sample shows characteristics of the family of ACC phases, which contain only the ν1 symmetric (1,085 cm^-1^) CO_3_^2-^ peak [15,18]. Note that there is an additional intense band at around 1,008 cm^-1^, which corresponds to the Si-(OH) stretching vibration of silica gel [19,20]. As a result, we can draw the conclusion that the branched sample is composed of silica gel and the ACC phase.

**Figure 3:**
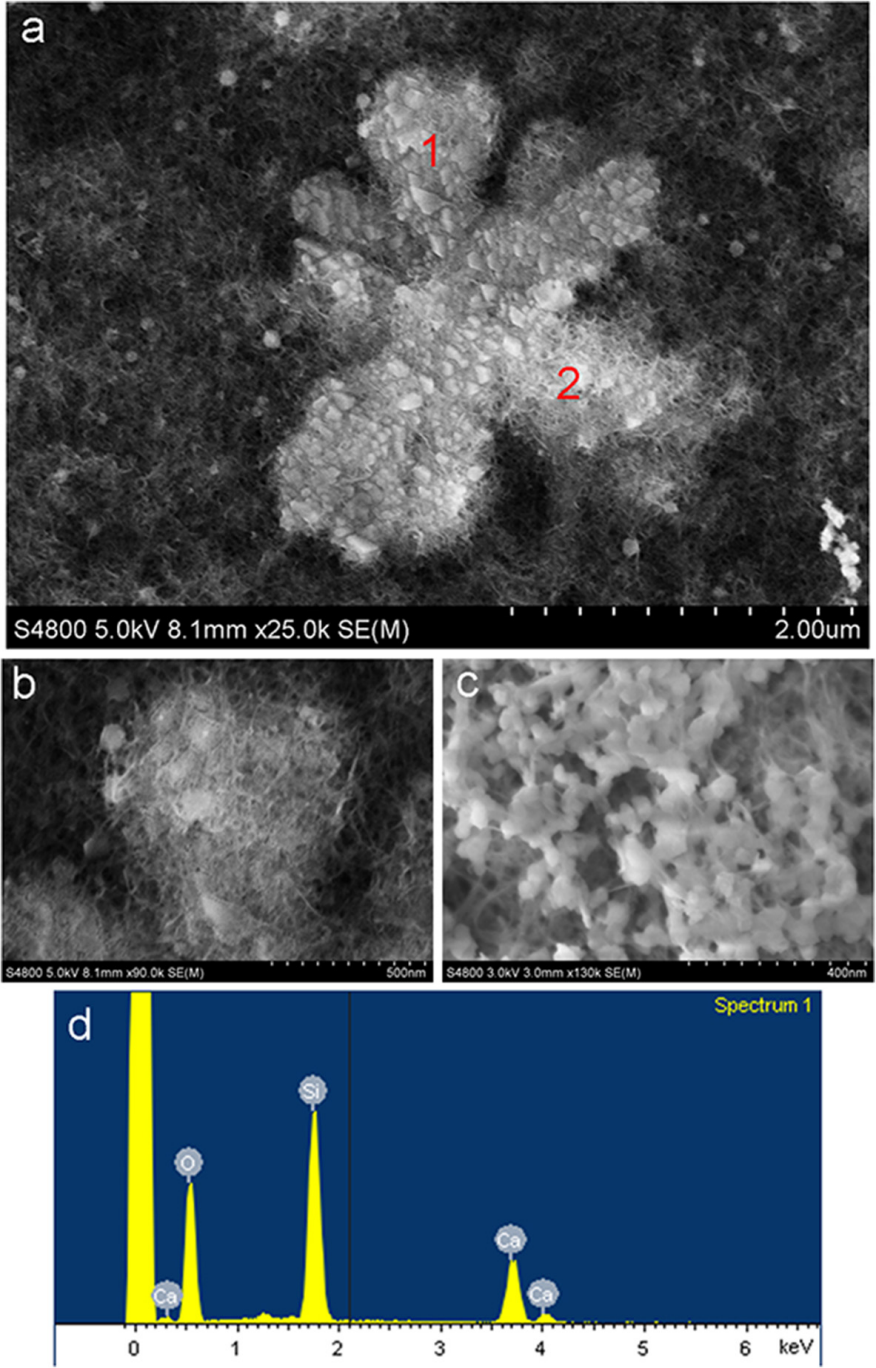
**SEM images. (a)** product with flower-like structure; **(b)** area 1 of **(a)** with high magnification; **(c)** area 2 of **(a)** with high magnification; **(d)** EDS spectrum of obtained flower-like product.

To investigate the nanostructure of the branched products, SEM was performed on a well-chosen flower-like product of sufficiently small size (Figure 3a). Figure 3b and Figure 3c are the magnified images, respectively, obtained from areas 1 and 2 of the flower-like product shown in Figure 3a. A fibrous matrix overspreads the field of view, and the flower-like crystallite is composed of a fibrous matrix and nanoparticles with a diameter of about 50 nm. The composition of the flower-like product was confirmed by EDS spectrum measurements (Figure 3d) to contain Si, O, and Ca, which reveals the existence of SiO_2_ and CaCO_3_. As C is a light element, it cannot be detected by EDS. Considering both the Raman results (Figure 2) and the SEM images (Figure 3), the branched samples were shown to consist of ACC nanoparticles and mesoporous silica gel. Namely, the structure of the resulting silica gel was successfully tailored to be mesoporous, which allows the existence of a stable ACC phase.

The ACC phase is usually the transient precursor of calcite [1], vaterite [2], or aragonite [4]. It is difficult to obtain stable ACC in the laboratory because of the large interfacial energy.

**Figure 4:**
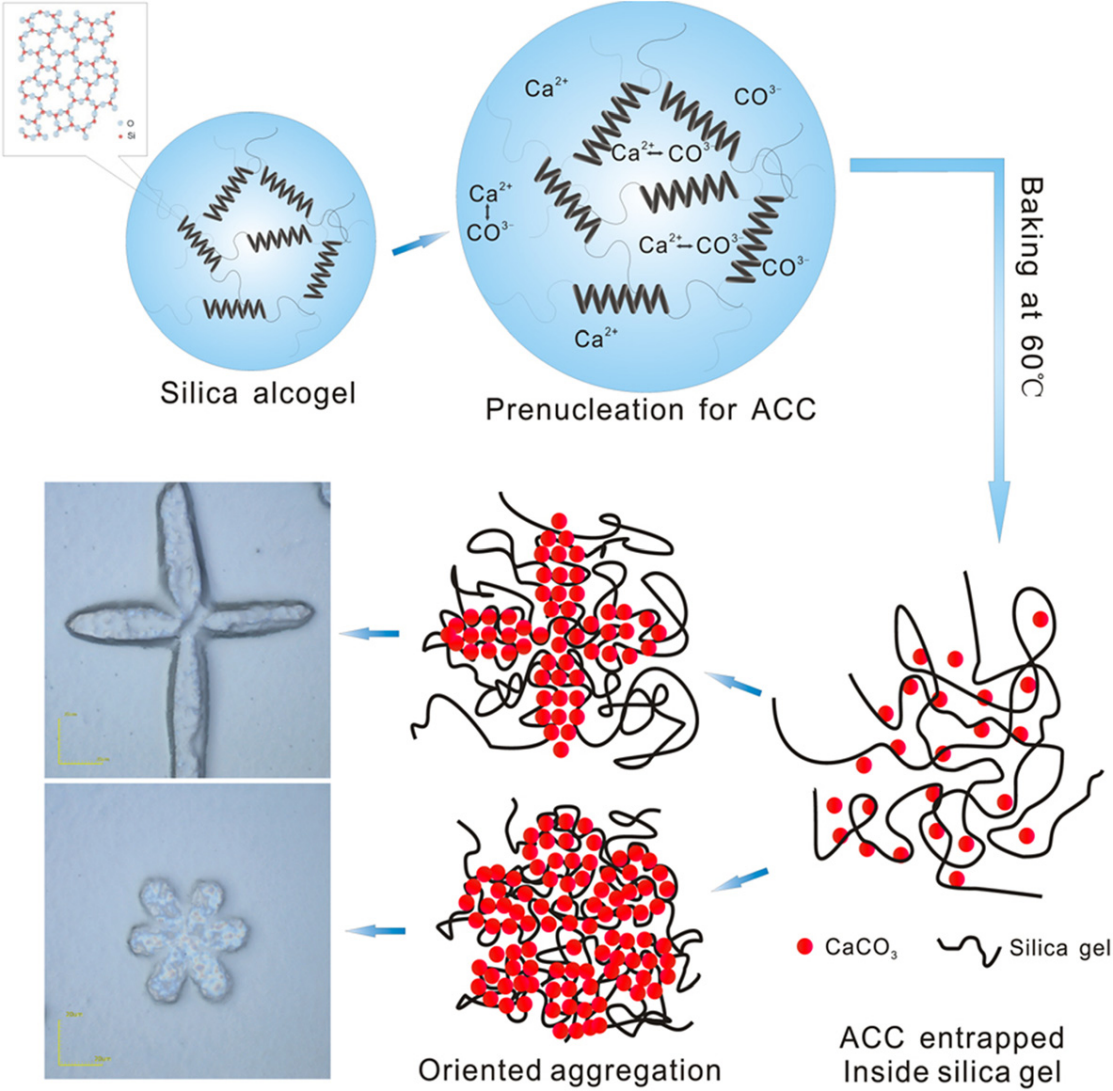
Schematic of possible self-assembly process for branched products.

According to the LSCM and SEM observations, a possible self-assembly process for the branched products is proposed, as illustrated in Figure 4. First, the hydrolysis and polycondensation reactions of ethyl silicate in an alkaline medium result in silica alcogel after stirring for 1 h. Second, by adding CaCl_2_ and urea solutions, ion pairs of Ca^2+^ and CO^3-^, which are prenucleation clusters for ACC aggregation [21-23], are formed in the solution. Third, when the mixed solution of CaCl_2_, urea, and, the silica alcogel is dried under a mild thermal treatment, namely, baking at 60°C, a mesoporous gel is obtained [9] and at the same time, ACC aggregates are entrapped in the silica gel voids, which has been demonstrated by SEM observation of the composites of fibrous silica gel and ACC (Figure 3). The mesoporous silica supports lower the ACC interfacial energy so that a composite of mesoporous silica gel and stable ACC is formed. Moreover, it can be seen that the ACC nanoparticles are aggregated in an oriented fashion so the branched morphology of the composites appears, as shown in Figure 1.

## Conclusions

In this work, the possibility of synthesizing stable ACC supported by mesoporous silica gel has been described. These composites are obtained using the reaction of CaCl_2_ and (NH_2_)_2_CO in a silica gel medium that is prepared through the hydrolytic polycondensation of ethyl silicate. LSCM, Raman, and SEM observations show that the morphology of the composites, which are composed of ACC nanoparticles and mesoporous silica gel takes on a branched form with cruciform-like and flower-like structures. The growth mechanism is discussed and a possible self-assembly process for the branched products is proposed. Silica gel with 3D-matrix morphology was successfully fabricated as a support for ACC. As a result, chemical agents with 3D-matrix morphology, such as silica gel, have the potential to significantly improve the utility and integrity of underground reservoirs for ACC storage. Moreover, the results suggest potential applications of the work with important implications in crystal engineering, biomimetic synthesis, etc.

## Competing interests

The authors declare that they have no competing interests.

## Authors' contributions

FF carried out the synthesis process of the composites, performed the statistical analysis, and drafted the manuscript. LGT and SX participated in the design of the study. XGX conceived of the study and participated in its design and coordination. XBH helped to draft the manuscript. All authors read and approved the final manuscript.

## Supplementary Material

Additional file 1: Figure S1 LSCM images (a) the products obtained from 2.5 mM CaCl_2._ (b) the products obtained from 10 mM CaCl_2_. Figure S1 shows the LSCM images of products grown in the mixing solutions with CaCl_2_ concentrations of 2.5 mM (Figure S1a) and 10 mM (Figure S1b) respectively. The regular branched products could not be found in Figure S1, which means no such branched products are formed with CaCl2 concentration which is lower than 5 mM or higher than 7.5 mM. Click here for file
