# Supplementary figures and images for: Synthesis of stable ACC using mesoporous silica gel as a support
Source: Nanoscale Res Lett. 2014 Aug 29;9(1):450. doi: 10.1186/1556-276X-9-450 (PMC4158389; doi:10.1186/1556-276X-9-450)

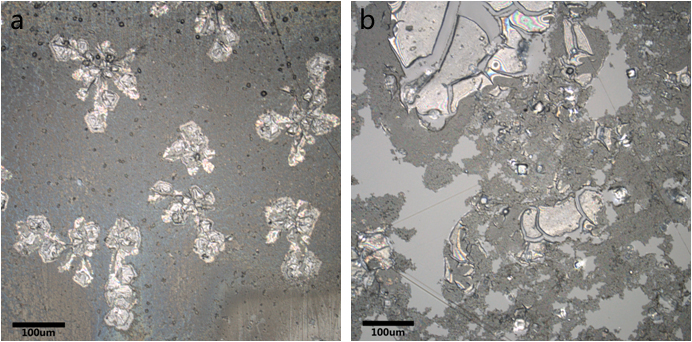

Supplement: Additional file 1: Figure S1 LSCM images — (a) the products obtained from 2.5 mM CaCl2. (b) the products obtained from 10 mM CaCl2. Figure S1 shows the LSCM images of products grown in the mixing solutions with CaCl2 concentrations of 2.5 mM (Figure S1a) and 10 mM (Figure S1b) respectively. The regular branched products could not be found in Figure S1, which means no such branched products are formed with CaCl2 concentration which is lower than 5 mM or higher than 7.5 mM. [file 1556-276X-9-450-S1.jpeg]
